# Beneficial adjunctive effects of the 5HT3 receptor antagonist ondansetron on symptoms, function and cognition in early phase schizophrenia in a double-blind, 2 × 2 factorial design, randomised controlled comparison with simvastatin

**DOI:** 10.1177/02698811241267836

**Published:** 2024-09-05

**Authors:** Imran B Chaudhry, Muhammad Omair Husain, Ameer B Khoso, Tayyeba Kiran, Muhammad Ishrat Husain, Inti Qurashi, Raza Ur Rahman, Nasir Mehmood, Richard Drake, Nusrat Husain, Bill Deakin

**Affiliations:** 1 Department of Psychiatry, Dow University of Health Sciences, Karachi, Pakistan; 2 Ziauddin University Hospital, Karachi, Pakistan; 3 Pakistan Institute of Living and Learning, Karachi, Pakistan; 4 Division of Psychology and Mental Health, School of Health Sciences, University of Manchester, Manchester, UK; 5 Campbell Family Mental Health Research Institute, Centre for Addiction and Mental Health, Toronto, ON, Canada; 6 Temerty Faculty of Medicine, Department of Psychiatry, University of Toronto, Toronto, ON, Canada; 7 Faculty of Health and Life Sciences, University of Liverpool, Merseyside, UK; 8 Manchester Academic Health Science Centre, Manchester, UK; 9 Mersey Care NHS Foundation Trust, Liverpool, Merseyside, UK

**Keywords:** Ondansetron, simvastatin, schizophrenia, clinical trial, negative symptoms

## Abstract

**Background:**

Variable benefits have been reported from the adjunctive use of simvastatin and the 5HT3 receptor antagonist, ondansetron, in patients with schizophrenia. We investigated their independent efficacy and possible synergy to improve negative symptoms of schizophrenia within a single trial.

**Methods:**

A 6-month, randomised, double-blind, placebo-controlled trial with a 4-arm, 2 × 2 factorial design, in three centres in Pakistan. In total, 303 people with stable treated schizophrenia aged 18–65 were randomly allocated to add-on ondansetron, simvastatin, both or neither. The primary outcome was a Positive and Negative Syndrome Scale (PANSS) negative score at 3 and 6 months.

**Results:**

Mixed model analysis and analysis of covariance revealed no main effects of simvastatin or ondansetron but a significant negative interaction between them (*p* = 0.03); when given alone, both drugs significantly reduced negative symptoms compared to placebo but they were ineffective in combination. Individual treatment effects versus placebo were −1.9 points (95%CIs −3.23, −0.49; *p* = 0.01) for simvastatin and −1.6 points for ondansetron (95%CIs −3.00, −0.14; *p* = 0.03). Combined treatment significantly increased depression and side effects. In those with less than the median 5 years of treatment, ondansetron improved all PANSS subscales, global functioning measures and verbal learning and fluency, whereas simvastatin did not.

**Conclusion:**

Small improvement in negative symptoms on simvastatin and ondansetron individually are not synergistic in combination in treating negative symptoms of schizophrenia. Ondansetron showed broad efficacy in patients on stable antipsychotic treatment within 5 years of illness. The findings suggest that ondansetron should be evaluated in patients at risk of psychosis or early in treatment.

## Introduction

This trial was carried out in response to a Stanley Medical Research Institute (SMRI) initiative for clinical trials repurposing drugs from a panel with potential anti-inflammatory actions that might benefit schizophrenia. We chose an efficient 2 × 2 factorial design that enabled us to simultaneously trial two unrelated drugs from the panel, ondansetron, which had shown benefit in a previous SMRI-funded trial (Zhang et al., 2006), and simvastatin, previously untested. The factorial design also allowed the detection of possible but unpredicted additive or synergistic effects of the very different primary and anti-inflammatory actions of the two drugs. At the time of the trial, non-steroidal anti-inflammatory drugs had shown promise as adjuncts to schizophrenia but there had been no trials of statins in schizophrenia and two had reported improved negative symptoms on ondansetron (Akhondzadeh et al., 2009; Sommer et al., 2012; Zhang et al., 2006).

HMG-CoA reductase inhibitors were known to have anti-inflammatory effects, in addition to their clinical lipostatic actions, for example, clinical efficacy in rheumatoid arthritis and promise as neuroprotective agents in neurodegenerative disorders (McCarey et al., 2004; Schönbeck et al., 2004; Sierra et al., 2011). Subsequent clinical trials in cardiovascular and autoimmune disorders, including multiple sclerosis, have demonstrated that the use of statins leads to a reduction in markers of inflammation and this correlates with improved outcomes (Sakata et al., 2019; Tang et al., 2011). A large epidemiological study showed that people with schizophrenia during treatment with statins had lower rates of psychiatric hospitalisation and self-harm (Hayes et al., 2019). These findings provided additional post-hoc support for evaluating statins in schizophrenia.

Ondansetron is a 5HT3 receptor antagonist currently used in the treatment of chemotherapy-induced nausea. However, experimental studies in rodents in the 1990s aroused great interest in the possible broad-spectrum efficacy of 5HT3 antagonists in schizophrenia, anxiety and cognitive enhancement (Costall, 1992). Initial interest was driven by preclinical effects involving modulation of neurotransmitter release including dopamine and acetylcholine (Engleman et al., 2008). Other studies reported the expression of 5HT3 receptors in immune cells including T cells and monocytes and anti-inflammatory effects through the inhibition of inflammatory cytokines such as tumour necrosis factor-alpha and interleukin-1-beta (Fiebich et al., 2004). Nevertheless, no trials were published until two small studies reported improvement in negative symptoms and one in cognition as an adjunct to treatment as usual (TAU) in schizophrenia (Akhondzadeh et al., 2009; Levkovitz et al., 2005; Zhang et al., 2006). This study aimed to replicate these findings.

We conducted a small, placebo-controlled, rater-blind 12-week pilot study adding ondansetron or simvastatin to TAU in 36 patients with chronic schizophrenia (Chaudhry et al., 2014). Both agents were well tolerated with no serious side effects. In the present study, the primary prediction was that the addition of ondansetron and/or simvastatin to TAU would improve negative symptoms. Secondary outcomes were improvements in positive symptoms, social functioning and cognitive functions. A preliminary analysis of this study was published in abstract form reporting small positive effects of both drugs versus placebo on negative symptom ratings when given alone but not when given in combination (Deakin et al., 2014). Here we present a full report together with our recent analyses revealing the mechanism of the negative interaction and the enhanced benefit of ondansetron in those with less than 5 years of drug treatment.

## Methods

### Study design and participants

We conducted a 6-month, double-blind placebo (P) controlled, factorial (2 × 2) design trial of ondansetron (OP) or simvastatin (SP) or both (OS) or neither (double placebo; PP) added to TAU in patients with schizophrenia, schizoaffective disorder, psychosis not otherwise specified and schizophreniform disorder. The trial was registered on Clinicaltrial.gov (NCT01602029) on 18 May 2012. The final protocol and statistical analysis plan were published (Chaudhry et al., 2013).

The study was conducted in Karachi, Pakistan. Participants were recruited from inpatient and outpatient psychiatric departments of the Dow University of Health Sciences, Karwan-e-Hayat Hospital and Abbasi Shaheed Hospital. Ethics approval for all sites was obtained from the ethics committee of the Pakistan Institute of Living and Learning (PILL/SMRI/1040) and the Dow University of Health Sciences (IBR-185/DUHS-10). Written informed consent was obtained from all participants prior to participation.

Participants between 18 and 65 years of age, meeting Diagnostic and Statistical Manual-IV (DSM-IV TR) diagnosis of schizophrenia, schizoaffective disorder, psychosis not otherwise specified or schizophreniform disorder were included. Participants were required to be stable on medication 4 weeks prior to enrolment with no planned medication changes. Female participants consented to adequate contraceptive measures and baseline and regular pregnancy tests. We excluded patients with organic brain disease, neurological diagnoses (including electroencephalogram conduction abnormalities, neurological disorder or an active seizure), and those meeting criteria for alcohol or substance abuse or dependence disorders.

Treating teams initially approached potential participants. A patient information leaflet with details of the study was given to potential participants and a meeting was arranged with the research team to explain the study in detail. At least 24 h was given prior to obtaining consent to participate in the study. Participants were informed that they were free to withdraw from the study at any time for any reason and that this would not impact their routine clinical care.

### Randomisation and masking

Participants were allocated according to a randomised permuted blocks algorithm generated by the trial statistician after stratification by the site. Only the trial pharmacist had access to treatment allocation for emergency unblinding authorised by the chief investigator or his deputy; unblinding did not prove necessary. Simvastatin was initiated at 20 mg once daily (OD), increasing to 40 mg after 4 weeks matched by placebo pills. Ondansetron was administered at 8 mg OD.

### Procedures

Clinical teams continued to provide usual care throughout the duration of the trial. Changes in medication were permissible, though stability was encouraged. Research assistants monitored the patients’ clinical state, side effects and pill compliance at week 2 and every 4 weeks until study completion. Efficacy endpoint assessments took place at 3 and 6 months. There were regular training and harmonisation sessions with reference to Positive and Negative Syndrome Scales (PANSS) interviews to sustain and monitor inter-rater reliability. The trial was monitored by an independent Trial Steering Committee (TSC) that included a senior physician and a service user. The TSC also had the responsibility for data monitoring to oversee any potential harm to the participants from taking part in the trial.

### Outcomes

The primary outcome measure was the negative syndrome subscale score on the PANSS rated 1–7 on seven items (Kay et al., 1987). Secondary symptomatic outcomes were as follows: Positive, General and Total PANSS scores; Calgary Depression Rating Scale (CDRS) rated 0–3 on nine items and Clinical Global Impression (CGI) rated 1 – normal to 7 – very severe. The functional outcomes were as follows: Global Assessment of Function Scale (GAF) (Hall 1995), 41–50 serious impairment, 91–100 no impairment; Social Functioning Scale (SFS) self-rating in 7 domains such as employment and recreation, 0–226 typically up to 135 and EuroQual Quality of Life Visual Analogue Scale (EQ-5D) 0–10 cm line (Addington et al., 2014; Birchwood et al., 1990; EuroQoL Group, 1990; Guy, 1976). Secondary cognitive performance measures included WAIS Block design; Stroop word/colour naming; Coughlan Verbal and Visual list learning; and Verbal Fluency (words and categories). Patients also completed a 30-item checklist of side effects based on the summary of product characteristics of ondansetron and simvastatin and the Antipsychotic Non-Neurological Side-Effects Rating Scale (ANNSERS) (Ohlsen et al., 2008).

### Power calculation and statistical analysis

The published power calculation was based on means and standard deviations from our separate pilot study (Chaudhry et al., 2013, 2014). The study statistician estimated that an analysis of variance of PANSS negative outcome scores from 43 participants per drug group would have 80% power to detect at *p* < 0.05 a 2-point superiority of pairwise comparisons of each treatment alone (OP and SP groups) over a final PANSS score of 20 in the double placebo group. The design had 45% power to detect an additional 1-point interactive benefit for the combined OS group. In the absence of significant interaction between simvastatin and ondansetron, the trial had over 90% power to detect the main effect of exposure to each drug whether alone or in combination, versus non-exposure. Thus, ondansetron exposure (OP + OS, *n* = 116) versus non-exposure (SP + PP, *n* = 117) gives the main effect of ondansetron with maximal sample sizes. Similarly, exposure to simvastatin given alone or in combination (PS + OS, *n* = 125) versus non-exposure (OP + PP, *n* = 108) gives the main effect of simvastatin. Assuming a drop-out rate of 20% (slightly worse than the feasibility rate), we aimed to recruit 54 participants per group.

Participants were assessed baseline and at the 3- and 6-month endpoints. Using liner mixed models (SPSS v27), we examined the main effects of simvastatin (yes or no) and ondansetron (yes or no), and of time (3 and 6 months) together with a random factor for participant effects. Baseline values of the outcome measures were covariates in the models. The two-way interaction between ondansetron and simvastatin was used to determine whether the two drugs showed synergistic interaction in their effects on outcome. Where justified by treatment interactions on outcome variables, pairwise comparisons of the four treatment groups were carried out with a four-level treatment factor as a fixed effect.

We assessed whether drug effects and their interaction differed at the 3- and 6-month endpoints by testing the two- and three-way interactions with time. In the absence of main or interacting effects of time, we estimated treatment effects common to the 3- and 6-month assessments. We tested two exploratory hypotheses – that the drugs might be more effective in those with a shorter illness duration, and influenced by the ongoing use of first- or second-generation antipsychotics. To simplify the analysis, we collapsed the 3- and 6-month ratings, removing the non-significant repeated time factor and adding a factor for the duration of illness or antipsychotic class in univariate ANOVAs of PANSS negative scores. Further analyses are reported in the text.

## Results

**Figure 1.**
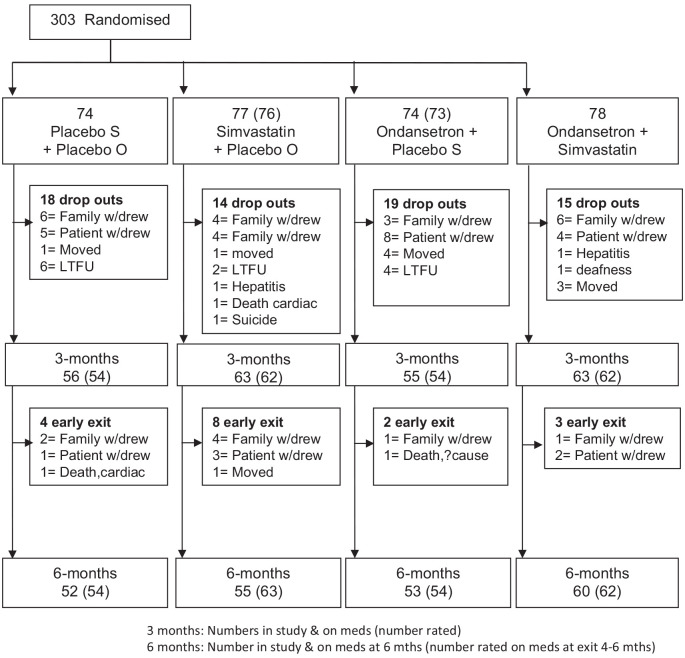
Patient flow through the study.

Participants were recruited between August 2010 and June 2013. We initially approached 426 participants and 24 were excluded with diagnoses of drug-induced psychosis. Of the 402 remaining who met inclusion criteria, 56 refused to participate in the study. The remaining 346 consented but 43 withdrew before baseline assessments were arranged. In total, 303 patients were randomised but 2 withdrew before baseline assessments (Figure 1). There were 74–78 patients in each treatment arm and 14–19 patients dropped out per arm for comparable reasons before the first assessment at 3 months, the majority (two-thirds) within 1 month.

There were no differences in demographic, clinical or functional measures between the completers and non-completers. A further 2–8 per arm left the study up to 2 months early but all but 4 completed final assessments that were included in the 6-month data. Thus, 77% of those randomised completed the study. Random allocation produced treatment arms with similar baseline characteristics although the combined treatment group had more participants of low socio-economic status (SES) than the other groups (Supplemental Table ST1). However, SES did not interact with treatment effects when added to the model. Similarly, there were no significant differences between the four groups in their usual antipsychotic, anticholinergic, antidepressant, anticonvulsant or lithium treatment at baseline (Supplemental Table ST1b).

There was no main effect of simvastatin or ondansetron on the primary outcome, PANSS negative symptoms (mean difference (95%CI) = −0.6 (−1.6, 0.37) *p* = 0.22 for simvastatin and −0.3 (−1.3, 0.7) *p* = 0.57 for ondansetron) (Supplemental Tables ST2). However, there was a statistically significant but negative overall interaction between ondansetron and simvastatin (*p* = 0.03). The pairwise follow-up comparisons revealed that patients receiving ondansetron alone or simvastatin alone showed greater improvements, respectively, of −1.5 and −1.9 points on PANSS negative scores than those receiving placebo (Table 1). By contrast, improvement in patients receiving both drugs was not statistically superior to improvement on placebo and was less, non-significantly, than the improvements seen with simvastatin or ondansetron alone. The results indicate that, despite their individual benefits, the two drugs did not add or synergise in reducing negative symptoms when taken in combination but rather weakened each other’s actions. There were no statistically significant main effects of ondansetron or simvastatin on secondary PANSS positive, general or total scores, on overall severity (CGI) or functional measures (SOFAS; SFS) (Supplemental Spreadsheet). There were no main or interacting effects of time on any outcome measure.

**Table 1.** Interaction of simvastatin and ondansetron on PANSS negative symptoms.

	Placebo + Placebo		Placebo + Simvastatin		Placebo + Ondansetron		Simvastatin + Ondansetron	
Time	Mean ± SEM	*N*	Mean ± SEM	*N*	Mean ± SEM	*N*	Mean ± SEM	*N*
Baseline	17.4 ± 0.76	74	18.0 ± 0.71	76	17.4 ± 0.71	73	17.7 ± 0.65	78
3 months	15.6 ± 0.74	54	13.4 ± 0.61	62	13.8 ± 0.68	54	14.1 ± 0.61	62
6 months	15.4 ± 0.86	54	14.2 ± 0.57	63	14.3 ± 0.67	54	14.6 ± 0.55	62
Estimated	15.5 ± 0.51	53	13.8 ± 0.47***	62	14.0 ± 0.51**	53	14.4 ± 0.47*	61

PANSS negative scores in the individual treatment groups at 3 and 6 months and estimated baseline-controlled marginal means are compared to show the interaction between ondansetron and simvastatin (*p* = 0.03). Exposure to simvastatin or ondansetron alone was significantly more effective than placebo–placebo in reducing PANSS negative symptom scores: *p ⩽ 0.1; **p ⩽ 0.05; ***p ⩽ 0.01; ****p ⩽ 0.005, whereas combined treatment was not. SEM=Standard Error of the Mean.

PANSS: Positive and Negative Syndrome Scales.

Calgary Depression Scale scores and side effect self-ratings were heavily skewed to zero and low scores with long positive tails that were not amendable or transformable to parametric mixed models analysis. The group medians and interquartile ranges are therefore shown for each of the four groups in Table 2. High CDRS depression scores at 6 months and high side effect ratings (ANNSERS and Total Side effects) at 3 months were more prevalent in those receiving both treatments than in the other three groups. This is reflected in the significant Kruskal–Wallis tests which may also reflect the contrasting shift to lower depression and side effect scores in those receiving either treatment alone compared to the placebo and the combined treatment groups.

**Table 2.** Calgary depression ratings and side effects increased with the combination of simvastatin and ondansetron treatment versus monotherapy.

Rating	Placebo + Placebo		Placebo + Simvastatin		Placebo + Ondansetron		Simvastatin + Ondansetron	
Time	Median [IQR]	*N*	Median [IQR]	*N*	Median [IQR]	*N*	Median [IQR]	*N*
Calgary Depression Rating Scale								
Baseline	4 [1, 6]	73	3 [1, 6]	76	3 [0, 6]	72	5 [1, 9]	77
3 months*	2 [0, 6]	54	2 [0, 4]	62	2 [0, 5]	54	3 [1, 8]	62
6 months****	2 [0, 5]	54	1 [0, 5]	63	2 [0, 5]	54	5 [1, 9]	62
ANNSERS								
Baseline*	7 [4, 12]	73	6 [3, 10]	76	7 [4, 12]	73	9 [5, 15]	77
3 months**	6 [4, 10]	54	5 [3, 8]	61	5 [2, 8]	54	7 [4, 12]	77
6 months	4 [2, 7]	54	4 [1, 8]	63	6 [2, 8]	54	6 [3, 9]	62
Total side effects								
Baseline	4 [1, 6]	71	2 [0, 5]	74	3 [1, 6]	70	3 [1, 7]	76
3 months****	2 [1, 4]	54	2 [0, 4]	62	2 [0, 3]	53	3 [2, 5]	60
6 months	1 [0, 3]	54	1 [0, 3]	63	1 [0, 3]	52	2 [0, 4]	60

Depression and side effect ratings are compared across the four individual treatment groups at baseline and follow-up. Kruskal–Wallis tests for significant deviation from chance distribution of rankings between the four groups at each follow-up are indicated by: *p ⩽ 0.1; **p ⩽ 0.05; ***p ⩽ 0.01; ****p ⩽ 0.005. Combined treatment was associated with greater depression ratings than other groups at 6 months and greater side effect ratings at 3 months.

ANNSERS: Antipsychotic non-neurological side effects rating scale; IQR: interquartile range.

We did not pursue paired group comparisons. Individually, of the 30 possible side effects, headache, depression and joint pains were significantly more prevalent in the combined treatment group than the other treatment groups at 3 months (Supplemental Table ST3).

**Figure 2.**
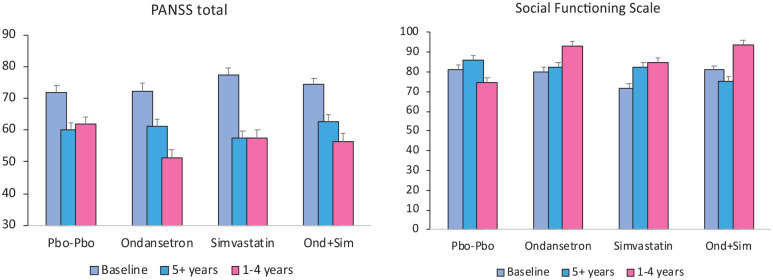
Ondansetron has a greater therapeutic effect in those with less than 5 years of illness. Grey histograms: Mean + SEM baseline scores PANSS total and Social Functioning Scores; blue and pink histograms are overall endpoint scores, respectively, of those with *above and below median durations of illness*. The ondansetron group improved significantly more than the Pbo-Pbo group in those with shorter durations of illness. No effect of treatment group in those with longer illnesses. The minimum score on PANSS total is 30. PANSS: Positive and Negative Syndrome Scale; Pbo: Placebo; Ondansetron: ondansetron + placebo simvastatin; Simvastatin: simvastatin + placebo ondansetron; Ond + Sim: ondansetron + simvastatin combination.

We investigated whether the small clinical effects shared by the two unrelated drugs in the total sample might indicate greater efficacy in subgroups responsive to their specific actions. We performed two exploratory analyses of covariance to determine whether drug effects depended differentially on previous duration and type of antipsychotic drug exposure, factors that are known to influence negative symptoms. Using a median split of <5 versus 5+ years of drug treatment, there was an ondansetron by duration interaction (*p* = 0.02) on PANSS negative symptoms suggesting efficacy in those with shorter treatment. We therefore re-ran the analysis of drug main effects and interactions on negative symptoms and other outcomes in those below the median of 5 years of treatment (Table 3). The ondansetron by simvastatin interaction on negative symptoms remained significant so we examined post hoc the pairwise therapeutic benefit of ondansetron alone (*n* = 27) which showed a 3-point difference from placebo (*n* = 27; 95% CIs 0.95, 5.199; *p* = 0.005). There were several statistically significant and trend level significant (*p* < 0.10) main effects of ondansetron on all PANSS symptoms, CGI and cognitive verbal learning and fluency (Table 3 and Figure 2). The significant main effects of ondansetron were accompanied by trends to negative interaction with simvastatin and, in view of the adverse effects of the combined group in the main analysis, we explored the efficacy of ondansetron alone and found greater effects than the main effects. Simvastatin did not interact with antipsychotic exposure and neither of the trial treatments interacted with first- versus second-generation antipsychotic class in influencing negative symptoms. However, PANSS positive, general and total scores improved minimally more on simvastatin versus no simvastatin in those receiving first-generation antipsychotics (*n* = 96) but not in those (*n* = 132) receiving second-generation drugs alone (significant simvastatin × antipsychotic drug class interactions all *p* < 0.05).

**Table 3.** Treatment effects of ondansetron and interaction with simvastatin on outcome measures in participants with less than 5 years of treatment.

Main effects of ondansetron versus no ondansetron					Interaction versus simvastatin
*Pairwise effect of ondansetron alone versus placebo*					
	Mean difference	SEM	*p* value	95% CIs	*p* value
**PANSS**					
Negative	1.66	0.73	0.026	0.20, 3.11	0.06
*Pairwise*	*3.07*	*1.07*	*0.005*	*0.95, 5.20*	
Positive	1.73	0.76	0.024	0.24, 3.23	0.13
*Pairwise*	*2.16*	*1.11*	*0.054*	*0.04, 4.35*	
General	2.97	1.24	0.018	0.51, 5.43	0.15
*Pairwise*	*4.75*	*1.83*	*0.011*	*1.13, 8.37*	
Total	6.25	2.40	0.010	1.50, 10.00	0.12
*Pairwise*	*10.05*	*3.52*	*0.005*	*3.07, 17.03*	
**Global function**					
CGI	0.44	0.17	0.012	0.10, 0.78	0.08
*Pairwise*	*0.75*	*0.25*	*0.004*	*0.25, 1.25*	
GAF	0.09	0.21	>0.10	−0.30, 0.49	
EQ-5D	6.24	0.36	0.069	−0.50, 12.99	0.03
*Pairwise*	*13.87*	*4.99*	*0.006*	*3.99, 23.75*	
SFS	12.90	5.36	0.018	2.26, 23.54	
**Cognitive**					
Stroop	9.34	14.7	>0.10	−19.79, 38.50	
Memory for words	5.30	2.21	0.018	0.92, 9.69	
Memory for designs	3.01	1.98	0.132	−0.92, 6.94	
Category fluency	2.01	0.93	0.033	0.16, 3.85	

Sample sizes: Main effects of ondansetron (OP + OS) *n* = 61 versus (PP + PS) *n* = 56. Pairwise Ondansetron alone (OP) *n* = 27 versus Placebo (PP) *n* = 27. Estimated means are shown in Supplemental Table ST4.

CGI: Clinical Global Impression; CI: confidence interval; EQ-5D: EuroQual Quality of Life; PANSS: Positive and Negative Syndrome Scales; SFS: Social Functioning Scale; SOFAS: GAF: Global Assessment of Function.

## Discussion

The primary objective of the study was to examine the efficacy of adjunctive simvastatin and ondansetron on negative symptoms of schizophrenia. Treatment with either simvastatin or ondansetron given alone was associated with small but statistically significant improvement in negative symptoms assessed at 3 and 6 months. Combined treatment with simvastatin and ondansetron did not demonstrate a synergistic effect but rather showed a statistically significant negative interaction with a weakened benefit on negative symptoms compared to individual administration. It appears probable that the negative therapeutic interaction is related to the increased depression ratings on the CDRS and greater experience of side effects in the combined group compared with the other treatments or placebo. Three specific side effects, headache, depression and joint pain, were over-represented in the combined group but they were not sufficiently serious to increase withdrawals from the combined group. A pharmacokinetic interaction increasing levels of one or other drug cannot be ruled out in the absence of drug levels but would not be predicted from their cytochrome profiles nor the absence of reports of clinically significant interaction. It is also possible that the benefit of one or both of the individual treatments reflects an increase in antipsychotic concentrations in plasma or brain but there is no obvious reason to predict this.

Since the completion of the study, five trials of statins have been reported, three with simvastatin and two with lovastatin or pravastatin with little evidence of benefit on negative symptoms. One study reported a benefit selectively on PANSS negative ratings of similar magnitude to our study (1.4 point difference) by 8 weeks in a population with a 20-year average duration of symptoms (Tajik-Esmaeeli et al., 2017). Weiser et al. (2023) recently reported trends of improvement in negative symptoms (*p* = 0.06) and withdrawal (*p* = 0.03) on simvastatin but these secondary outcomes did not survive correction for multiple comparisons. A third simvastatin study found no benefit on total PANSS or negative scores over 1 year of simvastatin in patients within 5 years of onset of psychosis (Sommer et al., 2021). Lovastatin (Ghanizadeh et al., 2014) and pravastatin (Vincenzi et al., 2014) showed no benefit on PANSS ratings. In a recent meta-analysis, which includes the data presented here, Weiser et al. (2023) found evidence of a small overall benefit of statins on total and positive symptoms but none on negative symptoms. However, the latter analysis excluded the positive result from Tajik-Esmaeeli et al. (2017) and included our main effect of simvastatin rather than the significant simvastatin alone data.

Our study was carried out in Pakistan which lacks comprehensive psychiatric support services and has a higher background exposure to potential infectious and nutritional components of immune pathogenesis. These factors might have accounted for the modest improvement of negative symptoms on simvastatin. However, the relative efficacy of simvastatin on PANSS scores in patients taking first-generation antipsychotic drugs might suggest the benefit derives in part from opposing an adverse possibly inflammatory component of the older drugs. Equally, high-dose simvastatin has neuroprotective actions as strikingly demonstrated by the prevention of grey matter loss and preservation of function in secondary progressive multiple sclerosis in the absence of changes in inflammatory markers (Chataway et al., 2014). The present effects and those reported are too small to warrant further exploratory studies until there are biomarkers for neurotoxicity or inflammation that simvastatin, the most brain-penetrant statin, could correct.

The most compelling finding is that adjunctive treatment with ondansetron appears to have efficacy on symptomatic, functional and cognitive domains of schizophrenia in patients with fewer than 5 years of antipsychotic treatment. Whether drug exposure, duration of illness or proximity to onset is the key factor cannot be disentangled. Four trials from an Iranian group employed a similar design with 20 stable participants per group with more than 10 years of illness and taking risperidone, two with ondansetron (Akhondzadeh et al., 2009; Samadi et al., 2017) and one each with granisetron (Khodaie-Ardakani, 2013) and tropisetron (Noroozian et al., 2013). They report 2–4 points of improvement on PANSS negative symptoms, none with improvement on positive, and some reporting improvement on total and general subscale scores. A large study in China of 121 treatment-resistant patients with a 17-year mean duration of illness and taking haloperidol (Zhang et al., 2006) reported improvement in PANSS negative symptoms (4 points) and total score (10 points) symptoms similar in magnitude to our findings in the short duration group but without changes in CGI or measures of cognitive performance. These findings were not replicated in a study in Australia of 85 patients also with long-standing treatment (Kulkarni et al., 2018). We appear to have detected greater benefits of ondansetron across a range of symptomatic, functional and cognitive outcomes because we were able to study a substantial subgroup with a short duration of drug exposure compared to previous studies. The profile of improvements in functional and cognitive domains suggests that 5HT3 antagonists may act to enhance cognition rather than reduce psychotic symptoms to improve function.

5HT3 receptors modulate the release of neurotransmitters including dopamine and acetylcholine from nerve terminals and are expressed by a sub-population of Gamma-amino butyric acid (GABA) interneurons (Puig et al., 2004). The receptors are fast-acting ligand-gated ion channels that influence cortical electrophysiological processes such as sensory gating that are thought to be impaired in schizophrenia. A recent review of 11 human studies, 8 in schizophrenia, found that 5HT3 antagonists improved sensory gating or visual sensory-motor processing (Tsitsipa et al., 2022). Such biomarkers may be a way to identify which patients might benefit from safe and inexpensive ondansetron therapy early in the evolution of schizophrenia.

A limitation of the present study was the inability to obtain more direct measures of inflammation such as cytokines which were not available at that time in Pakistan. Future studies should collect more comprehensive inflammatory biomarkers and T-cell immunophenotyping to identify at baseline individuals who are more likely to benefit from anti-inflammatory treatments. Another limitation is not standardising TAU; however, it would not have been feasible to standardise treatment for outpatients in the community in a trial of this size. Most participants were on second-generation antipsychotics and random allocation ensured there were no statistically significant differences in TAU across groups at baseline. The ondansetron analysis in the half-sample with less than 5 years antipsychotic exposure is exploratory, has less power than the main analysis and should clearly be treated with caution. However, it is noteworthy that the effects are not sporadic but consistent across all the main outcome measures. The 6-month duration of our study is a strength as most clinical trials of simvastatin and ondansetron have been limited by both small sample sizes and short duration of follow-up. Another important factor to consider when interpreting these results is that the study was conducted in Pakistan, a low and middle-income country where diet, nutritional status and other lifestyle factors differ from those in high-income countries. While these factors should be balanced among the groups in a randomised study with a reasonably large sample size, our findings may not be generalisable to high-income countries.

In conclusion, our study examined the potential benefits of two novel agents in the treatment of schizophrenia. Both drugs caused statistically significant but clinically unimportant reductions in negative symptoms, the primary outcome variable. Although it was hypothesised that the two agents could have additive effects, when taken in combination, their effects were lessened by the emergence of depressive symptoms and increased side effects. An exploratory analysis revealed a full spectrum of symptomatic, functional and cognitive improvement in patients with less than 5 years of treatment when exposed to ondansetron whether alone or in combination with simvastatin. However, the benefits of ondansetron were not clinically transformative in this stable population and although there might be greater benefit on persistent symptoms, the latter are best treated early with clozapine. We suggest that ondansetron might most usefully be evaluated in help-seeking patients with the prodromal at-risk mental state prior to the need for antipsychotic medication. Such patients have a broad range of perceptual, affective and cognitive symptoms, and functional impairment for which there is no indicated medical treatment and which we found benefitted from ondansetron, a well-tolerated and safe drug.

## Supplemental Material

sj-docx-1-jop-10.1177_02698811241267836 – Supplemental material for Beneficial adjunctive effects of the 5HT3 receptor antagonist ondansetron on symptoms, function and cognition in early phase schizophrenia in a double-blind, 2 × 2 factorial design, randomised controlled comparison with simvastatin Supplemental material, sj-docx-1-jop-10.1177_02698811241267836 for Beneficial adjunctive effects of the 5HT3 receptor antagonist ondansetron on symptoms, function and cognition in early phase schizophrenia in a double-blind, 2 × 2 factorial design, randomised controlled comparison with simvastatin by Imran B Chaudhry, Muhammad Omair Husain, Ameer B Khoso, Tayyeba Kiran, Muhammad Ishrat Husain, Inti Qurashi, Raza Ur Rahman, Nasir Mehmood, Richard Drake, Nusrat Husain and Bill Deakin in Journal of Psychopharmacology

sj-docx-2-jop-10.1177_02698811241267836 – Supplemental material for Beneficial adjunctive effects of the 5HT3 receptor antagonist ondansetron on symptoms, function and cognition in early phase schizophrenia in a double-blind, 2 × 2 factorial design, randomised controlled comparison with simvastatin Supplemental material, sj-docx-2-jop-10.1177_02698811241267836 for Beneficial adjunctive effects of the 5HT3 receptor antagonist ondansetron on symptoms, function and cognition in early phase schizophrenia in a double-blind, 2 × 2 factorial design, randomised controlled comparison with simvastatin by Imran B Chaudhry, Muhammad Omair Husain, Ameer B Khoso, Tayyeba Kiran, Muhammad Ishrat Husain, Inti Qurashi, Raza Ur Rahman, Nasir Mehmood, Richard Drake, Nusrat Husain and Bill Deakin in Journal of Psychopharmacology

sj-docx-3-jop-10.1177_02698811241267836 – Supplemental material for Beneficial adjunctive effects of the 5HT3 receptor antagonist ondansetron on symptoms, function and cognition in early phase schizophrenia in a double-blind, 2 × 2 factorial design, randomised controlled comparison with simvastatin Supplemental material, sj-docx-3-jop-10.1177_02698811241267836 for Beneficial adjunctive effects of the 5HT3 receptor antagonist ondansetron on symptoms, function and cognition in early phase schizophrenia in a double-blind, 2 × 2 factorial design, randomised controlled comparison with simvastatin by Imran B Chaudhry, Muhammad Omair Husain, Ameer B Khoso, Tayyeba Kiran, Muhammad Ishrat Husain, Inti Qurashi, Raza Ur Rahman, Nasir Mehmood, Richard Drake, Nusrat Husain and Bill Deakin in Journal of Psychopharmacology

sj-docx-4-jop-10.1177_02698811241267836 – Supplemental material for Beneficial adjunctive effects of the 5HT3 receptor antagonist ondansetron on symptoms, function and cognition in early phase schizophrenia in a double-blind, 2 × 2 factorial design, randomised controlled comparison with simvastatin Supplemental material, sj-docx-4-jop-10.1177_02698811241267836 for Beneficial adjunctive effects of the 5HT3 receptor antagonist ondansetron on symptoms, function and cognition in early phase schizophrenia in a double-blind, 2 × 2 factorial design, randomised controlled comparison with simvastatin by Imran B Chaudhry, Muhammad Omair Husain, Ameer B Khoso, Tayyeba Kiran, Muhammad Ishrat Husain, Inti Qurashi, Raza Ur Rahman, Nasir Mehmood, Richard Drake, Nusrat Husain and Bill Deakin in Journal of Psychopharmacology

sj-docx-5-jop-10.1177_02698811241267836 – Supplemental material for Beneficial adjunctive effects of the 5HT3 receptor antagonist ondansetron on symptoms, function and cognition in early phase schizophrenia in a double-blind, 2 × 2 factorial design, randomised controlled comparison with simvastatin Supplemental material, sj-docx-5-jop-10.1177_02698811241267836 for Beneficial adjunctive effects of the 5HT3 receptor antagonist ondansetron on symptoms, function and cognition in early phase schizophrenia in a double-blind, 2 × 2 factorial design, randomised controlled comparison with simvastatin by Imran B Chaudhry, Muhammad Omair Husain, Ameer B Khoso, Tayyeba Kiran, Muhammad Ishrat Husain, Inti Qurashi, Raza Ur Rahman, Nasir Mehmood, Richard Drake, Nusrat Husain and Bill Deakin in Journal of Psychopharmacology

sj-xlsx-6-jop-10.1177_02698811241267836 – Supplemental material for Beneficial adjunctive effects of the 5HT3 receptor antagonist ondansetron on symptoms, function and cognition in early phase schizophrenia in a double-blind, 2 × 2 factorial design, randomised controlled comparison with simvastatin Supplemental material, sj-xlsx-6-jop-10.1177_02698811241267836 for Beneficial adjunctive effects of the 5HT3 receptor antagonist ondansetron on symptoms, function and cognition in early phase schizophrenia in a double-blind, 2 × 2 factorial design, randomised controlled comparison with simvastatin by Imran B Chaudhry, Muhammad Omair Husain, Ameer B Khoso, Tayyeba Kiran, Muhammad Ishrat Husain, Inti Qurashi, Raza Ur Rahman, Nasir Mehmood, Richard Drake, Nusrat Husain and Bill Deakin in Journal of Psychopharmacology
