# Supplementary material for: Beneficial adjunctive effects of the 5HT3 receptor antagonist ondansetron on symptoms, function and cognition in early phase schizophrenia in a double-blind, 2 × 2 factorial design, randomised controlled comparison with simvastatin
Source: J Psychopharmacol. 2024 Sep 5;38(9):818–26. doi: 10.1177/02698811241267836 (PMC11445972; doi:10.1177/02698811241267836)
Supplement: sj-docx-1-jop-10.1177_02698811241267836 – Supplemental material for Beneficial adjunctive effects of the 5HT3 receptor antagonist ondansetron on symptoms, function and cognition in early phase schizophrenia in a double-blind, 2 × 2 factorial design, randomised controlled comparison with simvastatin [file sj-docx-1-jop-10.1177_02698811241267836.docx]

|  | Placebo | Simvastatin | Placebo + | Simvastatin + |
| --- | --- | --- | --- | --- |
|  | + Placebo | + Placebo | Ondansetron | Ondansetron |
| Number | 74 | 76 | 74 | 78 |
|  |  |  |  |  |
| Male sex n (%) | 41 (55) | 40 (52) | 52 (70) | 46 (59) |
|  |  |  |  |  |
| Age, mean (SD) | 32.9 (9.36) | 32.7 (9.16) | 32.2 (9.65) | 32.1 (8.79) |
|  |  |  |  |  |
| Marital Status (n) |  |  |  |  |
| Single | 48 | 47 | 51 | 46 |
| Married | 24 | 23 | 20 | 28 |
| Divorced | 1 | 5 | 3 | 3 |
| Widowed | 1 | 1 | 0 | 1 |
|  |  |  |  |  |
| Education, mean yrs (SD) | 7.89 (4.22) | 6.63 (4.55) | 7.76 (4.15) | 6.13 (4.52) |
|  |  |  |  |  |
| Socioeconomic Class (n) |  |  |  |  |
| Lower | 21 | 37 | 41 | 49 |
| Lower middle | 15 | 11 | 15 | 12 |
| Middle | 38 | 28 | 17 | 17 |
| Upper Middle | 0 | 0 | 1 | 0 |

**Supplementary Table ST1**

**Demographic details**
