# Supplementary material for: Beneficial adjunctive effects of the 5HT3 receptor antagonist ondansetron on symptoms, function and cognition in early phase schizophrenia in a double-blind, 2 × 2 factorial design, randomised controlled comparison with simvastatin
Source: J Psychopharmacol. 2024 Sep 5;38(9):818–26. doi: 10.1177/02698811241267836 (PMC11445972; doi:10.1177/02698811241267836)
Supplement: sj-docx-2-jop-10.1177_02698811241267836 – Supplemental material for Beneficial adjunctive effects of the 5HT3 receptor antagonist ondansetron on symptoms, function and cognition in early phase schizophrenia in a double-blind, 2 × 2 factorial design, randomised controlled comparison with simvastatin [file sj-docx-2-jop-10.1177_02698811241267836.docx]

|  | Total participants (n=303) | | Less than 5yrs  (n=145) | |
| --- | --- | --- | --- | --- |
|  | n | % | n | % |
| Antipsychotics |  |  |  |  |
| 2nd generation | 169 | 56 | 85 | 59 |
| 1st generation | 73 | 24 | 37 | 26 |
| 1+2 generation | 48 | 16 | 17 | 12 |
| Anticholinergic | 229 | 76 | 110 | 76 |
| Antidepressants | 26 | 9 | 13 | 9 |
| Valproate | 90 | 30 | 35 | 24 |
| Lithium | 17 | 6 | 2 | 1 |
| Benzodiazepine | 130 | 43 | 62 | 43 |

**Supplementary Table ST1b**

**Baseline drug treatments**

*No statistically significant differences in proportion of each drug class across the 4 treatment groups by separate chi-square tests for each class. Note similar percentage use in those with less than 5 years of drug treatment compared to use in the overall group.*

|  | Total participants (n=303) | |
| --- | --- | --- |
|  | n | % |
| Risperidone | 120 | 40 |
| Haloperidol | 59 | 19 |
| Olanzapine | 57 | 19 |
| Quetiapine | 35 | 12 |
| Fluphenazine | 33 | 11 |
| Clozapine | 15 | 5 |
| Trifluperazine | 15 | 5 |
| Aripiprazole | 7 | 2 |
| Ziprazidone | 5 | 2 |
| Chlorpromazine | 2 | 1 |
| Anticholinergic | 229 | 76 |

**Use of individual antipsychotic drugs**
