# Supplementary material for: Beneficial adjunctive effects of the 5HT3 receptor antagonist ondansetron on symptoms, function and cognition in early phase schizophrenia in a double-blind, 2 × 2 factorial design, randomised controlled comparison with simvastatin
Source: J Psychopharmacol. 2024 Sep 5;38(9):818–26. doi: 10.1177/02698811241267836 (PMC11445972; doi:10.1177/02698811241267836)
Supplement: sj-docx-3-jop-10.1177_02698811241267836 – Supplemental material for Beneficial adjunctive effects of the 5HT3 receptor antagonist ondansetron on symptoms, function and cognition in early phase schizophrenia in a double-blind, 2 × 2 factorial design, randomised controlled comparison with simvastatin [file sj-docx-3-jop-10.1177_02698811241267836.docx]

**Supplementary Table ST2**

**Main effects of Simvastatin and Ondansetron on PANSS Negative symptoms**

| Timepoint | No Simvastatin | | Simvastatin | | Difference ^(*)^ | P-value |
| --- | --- | --- | --- | --- | --- | --- |
|  | Mean ± SD | N | Mean ± SD | N | Mean (95% CI) |  |
|  |  |  |  |  |  |  |
| Baseline | 17.4 ± 6.2 | 147 | 17.8 ± 5.9 | 154 |  |  |
| 3 months | 14.7 ± 5.3 | 108 | 13.8 ± 4.8 | 124 |  |  |
| 6 months | 14.9 ± 5.4 | 108 | 14.4 ± 4.5 | 125 |  |  |
| *Overall effect* | *14.8 ± 4.0* | *106* | *14.1± 3.4* | *123* | *-0.6 (-1.6, 0.37)* | *0.22* |
|  | . | | | |  |  |
|  | No Ondansetron | | Ondansetron | | Difference ^(*)^ |  |
|  | Mean ± SD | N | Mean ± SD | N | Mean (95% CI) |  |
|  |  |  |  |  |  |  |
| Baseline | 17.7 ± 6.3 | 150 | 17.5 ± 5.8 | 151 |  |  |
| 3 months | 14.4 ± 5.2 | 116 | 14.0 ± 4.9 | 116 |  |  |
| 6 months | 14.8 ± 5.4 | 117 | 14.4 ± 4.6 | 116 |  |  |
| *Overall effect* | *14.6 ± 3.8* | *115* | *14.3 ± 3.7* | *114* | *-0.3 (-1.3, 0.7)* | *0.54* |

*Legend to table 1.*

*The main effect of ondansetron combines the groups who are were exposed to ondansetron alone or in combination, the OP + OS groups, and compares them with those not exposed to ondansetron in the SP + PP groups. Similarly exposure to simvastatin occurred in the PS + OS groups while those in the OP + PP groups did not experience simvastatin.*
