# Supplementary material for: Beneficial adjunctive effects of the 5HT3 receptor antagonist ondansetron on symptoms, function and cognition in early phase schizophrenia in a double-blind, 2 × 2 factorial design, randomised controlled comparison with simvastatin
Source: J Psychopharmacol. 2024 Sep 5;38(9):818–26. doi: 10.1177/02698811241267836 (PMC11445972; doi:10.1177/02698811241267836)
Supplement: sj-docx-4-jop-10.1177_02698811241267836 – Supplemental material for Beneficial adjunctive effects of the 5HT3 receptor antagonist ondansetron on symptoms, function and cognition in early phase schizophrenia in a double-blind, 2 × 2 factorial design, randomised controlled comparison with simvastatin [file sj-docx-4-jop-10.1177_02698811241267836.docx]

**Supplementary Table ST3**

**Individual side effect frequencies showing treatment group differences**

| Side effect | Total | Placebo  + Placebo | Simvastatin  + Placebo | Ondansetron  + Placebo | Ondansetron + Simvastatin | p All | p OS vs PP |
| --- | --- | --- | --- | --- | --- | --- | --- |
|  |  |  |  |  |  |  |  |
| Headache | 112 | 21 | 25 | 25 | 41 | 0.009 | 0.003 |
| Depression | 49 | 8 | 12 | 10 | 19 | 0.172 | 0.044 |
| Joint pain | 42 | 8 | 8 | 5 | 21 | 0.002 | 0.018 |
| Rated |  | 54 | 62 | 54 | 62 |  |  |

*ST3 legend*

*Number of participants endorsing side effects by group at 3 months. P values from chi square analysis.*
