# Supplementary material for: Beneficial adjunctive effects of the 5HT3 receptor antagonist ondansetron on symptoms, function and cognition in early phase schizophrenia in a double-blind, 2 × 2 factorial design, randomised controlled comparison with simvastatin
Source: J Psychopharmacol. 2024 Sep 5;38(9):818–26. doi: 10.1177/02698811241267836 (PMC11445972; doi:10.1177/02698811241267836)
Supplement: sj-docx-5-jop-10.1177_02698811241267836 – Supplemental material for Beneficial adjunctive effects of the 5HT3 receptor antagonist ondansetron on symptoms, function and cognition in early phase schizophrenia in a double-blind, 2 × 2 factorial design, randomised controlled comparison with simvastatin [file sj-docx-5-jop-10.1177_02698811241267836.docx]

**Supplementary Table ST4**

**Estimated marginal means for main effects of ondansetron in Table 3**

|  | No ondansetron (PP + SP), n=56 | | | | All ondansetron (OP + OS), n=61 | | | |  |
| --- | --- | --- | --- | --- | --- | --- | --- | --- | --- |
| **PANSS** | Mean | SEM | 95% CIs | | Mean | SEM | 95% CIs | | p value |
| Negative | 14.87 | 0.53 | 13.82 | 15.91 | 13.21 | 0.51 | 12.20 | 14.22 | 0.026 |
| Positive | 15.07 | 0.54 | 14.00 | 16.15 | 13.34 | 0.52 | 12.30 | 14.38 | 0.024 |
| General | 30.17 | 0.89 | 28.40 | 31.94 | 27.20 | 0.86 | 25.50 | 28.91 | 0.018 |
| Total | 59.99 | 1.73 | 56.57 | 63.41 | 53.75 | 1.66 | 50.45 | 57.04 | 0.010 |
|  |  |  |  |  |  |  |  |  |  |
| **Global function** |  |  |  |  |  |  |  |  |  |
| CGI | 3.03 | 0.13 | 2.79 | 3.28 | 2.60 | 0.12 | 2.36 | 2.83 | 0.012 |
| SOFAS | 5.05 | 0.14 | 4.77 | 5.34 | 4.96 | 0.14 | 4.69 | 5.23 | >0.10 |
| EQ5D | 54.44 | 2.45 | 49.59 | 59.29 | 60.68 | 2.36 | 56.01 | 65.35 | 0.069 |
| SFS | 79.22 | 3.80 | 71.69 | 86.76 | 92.12 | 3.78 | 84.61 | 99.63 | 0.018 |
|  |  |  |  |  |  |  |  |  |  |
| **Cognitive** |  |  |  |  |  |  |  |  |  |
| Stroop | 90.28 | 10.64 | 69.12 | 111.44 | 99.63 | 10.08 | 79.59 | 119.67 | >0.10 |
| Memory for words | 31.36 | 1.59 | 28.20 | 34.51 | 36.66 | 1.54 | 33.62 | 39.70 | 0.018 |
| Memry for designs | 17.12 | 1.43 | 14.28 | 19.96 | 20.13 | 1.37 | 17.42 | 22.84 | 0.132 |
| Category fluency | 17.06 | 0.67 | 15.73 | 18.38 | 19.06 | 0.65 | 17.79 | 20.34 | 0.033 |
